# Implementing a stabilizing intervention for traumatized refugees in temporary accommodations in South-West Germany - a randomized controlled pilot trial

**DOI:** 10.3389/fpsyt.2024.1453957

**Published:** 2024-10-31

**Authors:** Irja Rzepka, Catharina Zehetmair, Ede Nagy, Hans-Christoph Friederich, Christoph Nikendei

**Affiliations:** Departement for General Internal and Psychosomatic Medicine, University Hospital Heidelberg, Heidelberg, Germany

**Keywords:** refugees, guided imagery techniques, ptsd, temporary accommodations, mental health care provision

## Abstract

The prevalence of trauma-related disorders, such as post-traumatic stress disorder (PTSD), as well as depressive or anxiety disorders, are significantly increased in refugees compared to the general population. This is due to the high risk of burdening and potentially traumatic experiences before, during and after their flight. However, the treatment options for these mental illnesses are significantly limited due to the legal uncertainties surrounding the asylum procedure as well as language and cultural barriers. In this study, a randomized controlled pilot trial was conducted to assess the effectiveness of self-applied audio-based mindfulness and guided imagery techniques for refugees with symptoms of post-traumatic stress disorder living in temporary accommodations. The symptom burden of PTSD, depression, and anxiety symptoms, as well as perceived stress and emotional state was assessed using the PC-PTSD-5, PHQ-4, Self-Assessment Manikin, and Stress Thermometer. *N*=32 refugees were included in the intervention study, *n*=24 participants completed the study. The audio-based mindfulness and guided imagery techniques were presented to *n* =17 refugees in the intervention group between T1 and T2 over 4 weeks; *n*=15 refugees from the waiting list control group received the audio-files 6 weeks after follow-up. Efficacy was evaluated using a mixed analysis of variance. There were no significant changes in the symptoms of post-traumatic stress disorder, depression and anxiety disorder. Moreover, no changes were observed in the level of perceived stress or emotional state. Only few participants used the exercises in self-administered application during the study period. This was mainly due to the stressors of the post-migratory phase, such as job search or worries about residence status. Due to the self-administered nature of the intervention, common factors, such as therapeutic alliance or expectations regarding the intervention’s effectiveness, had minimal influence and did not contribute to the sustained use of the exercises. Additionally, regarding the life circumstances of refugees in temporary shelters, the intervention intensity may not have been sufficient to have a significant impact on the examined symptom burden. Small sample size and limited use of the exercises limit the reliability of the results.

**Clinical trial registration:**

https://drks.de/search/de/trial/DRKS00022862 Deutsches Register Klinischer Studien identifier, DRKS00022862.

## 1 Introduction

Refugees are a population group that experience high burden in terms of mental health. Pre-, peri, and postmigration stress factors contribute to a high prevalence of post-traumatic stress disorder (PTSD), depression, and anxiety disorders (1). Even though exposition-based interventions are recommended for the treatment of PTSD (2–4), they can be accompanied by considerable distress due to the confrontation with trauma-related stimuli (5). Additionally, provision of adequate psychological treatment for refugees is impeded due to various challenges. Uncertain residence status during the asylum process or temporary residence permits can lead to limited access to mental health care (6, 7) and massive psychological burden for refugees due to the constant fear of deportation (8). In Germany, in the early post-migration phase, only acute illnesses and pain are treated (9). In cases where a heightened need for protection exists, such as those involving trauma-related disorders, early intervention options may be considered. While care for trauma-related disorders is typically offered by psychosocial centers across the country, these facilities are often unable to meet the demand and frequently have extended waiting periods. Besides, additional care must first be approved by the authorities (6, 7). Further, the language barrier is one of the most relevant obstacles to overcome in order to ensure appropriate mental health treatment (10). In addition to the challenge of implementing therapeutic measures with a third person as translators (11), the provision of an interpreter for psychotherapeutic treatment is also dependent on the financing. Furthermore, different expectations of what is achievable or unattainable through psychotherapeutic treatment or different explanatory models for mental illnesses can make psychotherapeutic treatment more difficult (12). Establishing reliable and continual therapeutic support - a crucial requirement for the use of trauma-confrontative therapy (13, 14) - is frequently unfeasible in these conditions. In light of refugees’ precarious living conditions and the problem of developing a viable psychological care framework, a stabilizing therapeutic approach should be considered for the treatment of PTSD in refugee populations. According to the definition of Luise Reddemann, stabilizing interventions help to improve symptom control, emotion regulation, and encourage the development of new skills (15, 16). In psychodynamic trauma therapy, imagination exercises are a possible tool for stabilization, especially in the treatment of complex trauma-related disorders, such as the imagination of an “inner safe place”, which offers an inner space of security (17). In the inpatient context, Psychodynamic Imaginative Trauma Therapy (18), which includes different imagination exercises in all phases of treatment, has led to the reduction of post-traumatic symptoms and an improvement in the ability to self-soothe (19). Due to the potential of applying the imagination exercises to situations outside of the therapeutic setting, as well as resulting from clinical experience, an initial pilot study was conducted in the refugee context. In this pilot study, mindfulness based and guided imagery techniques were provided in a group setting for adult English-speaking refugees with post-traumatic stress disorder in a registration and reception centre in Germany (20). The stabilizing techniques provided in this pilot study included a breathing exercise, the “Inner safe place”, the “Tree exercise”, both developed by Luise Reddemann (21), and the “Body Scan”, originally by Jon Kabat-Zinn (22). Through the intervention, participants showed a significant reduction of symptom load of anxiety and improvement in emotional well-being (20). The participants indicated, that they felt more relaxed, were better able to handle rumination (23), and that they used the stabilization techniques on their own as well (20, 24). After this initial pilot study, the exercises were also made available as audio files through the psychosocial outpatient clinic in the registration centre. It was demonstrated that refugees used the exercises and found that they helped them manage their symptoms (24). However, the duration of stay in the registration centres lasts usually lasts between few days and few weeks or months. Therefore, they limited the regular participation of the group sessions (20). Refugees are often taken to temporary placements without prior notice, where they are placed during the assessment of the asylum application or in the event of a temporary suspension of deportation (ger. “Duldung”) (25). Furthermore, because of the disparities in language, the group offer was limited to participants who speak the same language. Therefore, the stabilization exercises ought to be made available in different languages for refugees living in temporary accommodations for autonomous, flexible application to reach a larger number of affected people. So far, there has been little preliminary work on self-help applications for refugees with trauma-related disorders. Mazulla et al. (2021) assessed a language free mobile app, based on cognitive behavioural (CBT) and acceptance therapy (ACT) techniques. The use of the app in conjunction with personal meetings, showed a significant reduction of trauma-related symptoms (26). An app developed by Röhr et al. (2013) was also based on elements of CBT, but showed no significant reduction in PTSD symptoms in the intervention group compared to the control group (27). By providing the imagination exercises in the form of audio files in multiple languages, the current study aimed at reaching refugees in temporary shelters suffering from symptoms of post-traumatic stress disorder (PTSD) at low-threshold. The independent and flexible format aims to make the intervention accessible to a larger number of refugees in their native language. A randomized controlled pilot trial was conducted to evaluate the effectiveness of these audio-based interventions in reducing PTSD symptoms. The study also explored the feasibility of delivering mental health support through low-cost, accessible methods in diverse linguistic and cultural contexts, with the goal of developing larger-scale interventions for displaced populations.

## 2 Materials and methods

### 2.1 Participants

Adult refugees accommodated in 10 temporary shelters in the State of Baden-Württemberg, Germany were recruited for participation in this study. At the start of the study, there were around 690 refugees living in the housing facilities in the Rhine-Neckar district, including children. However, these numbers are always fluctuate, as the facilities are temporary accommodation for people during their asylum process or with a limited residence permit. No more detailed information on age or origin was available. The people were contacted directly in their shelters by the study team and informed about the study in the respective language (German, English, French, Turkish, Arabic, Farsi, Serbian). They were asked if they were willing to participate in an initial screening with two short questionnaires about symptoms of mental health burden and potentially in a following intervention study if symptoms of PTSD were present. We were able to contact 190 refugees living in these shelters. The screening was conducted with the PC-PTSD-5 and the PHQ-4. The PC-PTSD-5 pertains to six potentially traumatic events (war, witnessing a murder or serious injury or death of another person, physical/sexual violence or abuse, death of a loved one by murder or suicide, severe accident or fire, natural disaster), with the present study adding the events of “torture” and “captivity” which can also occur in the context of flight. Responses to these events are dichotomous, allowing for a “Yes” or “No” answer. Subsequently, respondents are asked, also using “Yes” or “No” answers, about typical PTSD symptoms (nightmares, avoidance behaviour, hyperarousal, feelings of alienation, guilt) (28). If any of the traumatic events are marked with a “yes” response, a cut-off value of 3 out of 5 core symptoms provides the highest sensitivity for detecting PTSD. The questionnaire demonstrates high diagnostic accuracy with a sensitivity of 0.95 and specificity of 0.85 (28). The PHQ-4 consists of two subscales: the Patient Health Questionnaire-2 (PHQ-2) (29), which focuses on the core symptoms of depression using two items, and the Generalized Anxiety Disorder-2 (GAD-2) (30), which also comprises two items assessing anxiety symptoms. The questions, “Little interest or pleasure in doing things,” “Feeling down, depressed, or hopeless,” “Feeling nervous, anxious, or on edge,” and “Not being able to stop or control worrying,” are quantified by the responses: “Not at all,” “Several days,” “More than half the days,” and “Nearly every day” (31). The response “Not at all” corresponds to a score of 0, and “Nearly every day” corresponds to a score of 3. A maximum of 6 points can be scored for both depression and anxiety. The recommended cut-off values for the subscales are 3 points each for detecting depression (29) or anxiety (30). The PHQ-4 demonstrates good internal consistency (*α* = 0.83) and construct validity (31), and has proven robust when applied to refugees in different languages (32). Those who reached the cut-off score of the PC-PTSD for PTSD, had sufficient language skills in one of the available languages and were willing to participate in the study after clarification about the content of the study could be included. All participants gave written consent.

### 2.2 Intervention

For the purpose of the study, three mindfulness-based and guided imagery techniques (breathing exercise, the inner safe place, the body scan) were translated in the most common languages used by refugees in the initial reception centre by the time of the study planning phase (English, French, Turkish, Arabic, Farsi, Serbian). These exercises can also be assessed as audio files and in written online https://www.heidelbergerklinischestandards.de/buecher/buch/heidelberger-standarduebungen-zur-stabilisierung-von-traumatisierten-gefluechteten/. The first exercise (“Breathing) is a simple mindfulness practice. The focus is on cultivating mindful awareness of bodily changes and movements while breathing, without judging. Participants are instructed not to follow distracting thoughts but instead to redirect their attention back to their breathing as soon as they realized they are becoming distracted. The “Body Scan,” created by Jon Kabat-Zinn (22), is the second exercise. In this exercise, participants systematically direct their attention to different parts of their body, beginning at the top of their head, and ending at their feet. Similarly to the breathing exercise, participants are instructed to notice their sensation without judgment, and cultivate a non-judgmental awareness (33). Mindfulness based exercises can help to reduce avoidance behavior as well as alterations in mood, cognition and arousal (34). The “Inner Safe Place” is a guided imagery exercise designed to evoke feelings of safety and comfort. Participants are asked to use their imagination or past experiences to vividly picture a place where they feel safe. These positive emotions can be internalized with continued practice, enabling participants to call upon them when they’re feeling anxious and unsettled (21). This guided imagery exercise is intended to help individuals who have experienced traumatic events evoke a sense of safety and security within themselves, thereby reducing feelings of fear or helplessness. All exercises were provided to the participants as audio files, allowing them to practice independently using their mobile phones.

Participants were randomly assigned to the intervention group or waitlist control group. If couples or family members participated, they were randomized per household. Simple randomization was carried out through random numbers in SPSS (35) by a member of the study team. The participants were informed verbally about the group allocation at the time of the first appointment. The intervention included an initial meeting with the participant, which was conducted by a member of the study team with the help of an interpreter via telephone in the participants mother tongue. This included psychoeducation about the nature of post-traumatic stress disorder and its symptoms. Further, they received information about the above-mentioned stabilization techniques, and practiced them with the member of the study team. Afterwards, the audio files were transferred to the participants’ phones, so they could be used directly. After two weeks, another meeting with a member of the study team was scheduled to ask if participants were using the techniques, clarify question, and practice again together. The participants of the waitlist control group received the same intervention (psychoeducation and explanation about the exercises in their respective language, exercises as audio files on their phone) after the last data assessment (T3).

### 2.3 Outcome measures

The questionnaire survey comprised short questionnaires assessing symptoms of PTSD [PC-PTSD-5 (28)] as the primary outcome. Secondary outcome comprises symptom load of depression and anxiety [PHQ-4 (31)] as described above. Additionally the current level of stress was assessed with the stress thermometer, which is taken from the Refugee Health Screener (36). It measures the current perceived stress level on a scale from 0 (“everything is fine”) to 10 (“I feel worse than ever before”). The Stress Thermometer is used for general assessment of stress without inferring the presence of a specific disorder; however, a score of ≥ 5 indicates a potential impairment of mental health (37). The “Self Assessment Manikin” (SAM) (38) is a brief psychometric questionnaire designed to assess the current affective state across the dimensions of valence, arousal, and dominance. This is depicted using gender- and culture-neutral figures representing these dimensions in five levels. The non-verbal representation makes the questionnaire easy to understand (39) and suitable for respondents from diverse backgrounds (40). For the statistical evaluation of this questionnaire, the pictures were each rated with ascending numbers from 1-5. The SAM questionnaire has also been successfully used with refugees (20, 41). The survey was carried out before the intervention (T1), after 4 weeks (T2) of self-practice for the intervention group, or at the beginning (T1) and after (T2) 4 weeks of waiting time for the waitlist control group, respectively. Follow-up (FU) was assessed 6 weeks after T2. The hypothesis was as follows: the flexible and autonomous application of the mindfulness and imaginative stabilizing exercises provided in the participants language as audio files result in a reduction in post-traumatic symptoms in the intervention group compared to a waiting list control group.

### 2.4 Statistical methods

The data analysis was carried out using a mixed ANOVA. The data were analysed if complete data set (pre, post, follow-up) was available. The relevant assumptions for the ANOVA were checked beforehand. The data were first tested for normal distribution using the Shapiro-Wilk test. The homogeneity of the error variances between the groups was checked using the Levene test, while the homogeneity of the covariance matrices was ensured by using the box test. At the beginning of the study, it was assumed that 80% of refugees in temporary accommodation could be reached with the available languages. With an average prevalence of PTSD of 20-40% (42) among the residents of the accommodation and 60% consent to the study, a possible sample size of 66-132 was assumed. The effect size of mindfulness based and imaginative techniques as provided in this study is not known. Therefore, we were guided by mindfulness-based interventions, that show medium to large effect sizes (34). We computed the required sample size for the data analyses using the software G-Power (43). A sample size of *n*=28 was demonstrated to be sufficient for mixed ANOVA (repeated measures: within-between interactions, ANOVA approach) assuming a medium effect of f=25 with α=.05, 1−β =.81, number of groups =2, number of measurements =3, corr. among rep measures = 0.5, and non-sphericity correction ϵ=1. Drop-out rates for psychological interventions for refugees are around 20%, with even higher percentages for waitlist control groups (44). Therefore, a sample size of *n*=34 was aimed for in order to be able to show a medium effect. However, the aim was also to reach as many of the potential residents of the accommodation as possible. Participants from of the intervention group additionally filled out a questionnaire about the frequency and use of the exercises and their everyday life and the effect on their symptoms. This study was approved by Ethics Committee of the Heidelberg Medical Faculty under the reference S-640/2016. The trial was registered at Deutsches Register Klinischer Studien (trial registration number: DRKS00022862).

## 3 Results

**Figure 1:**
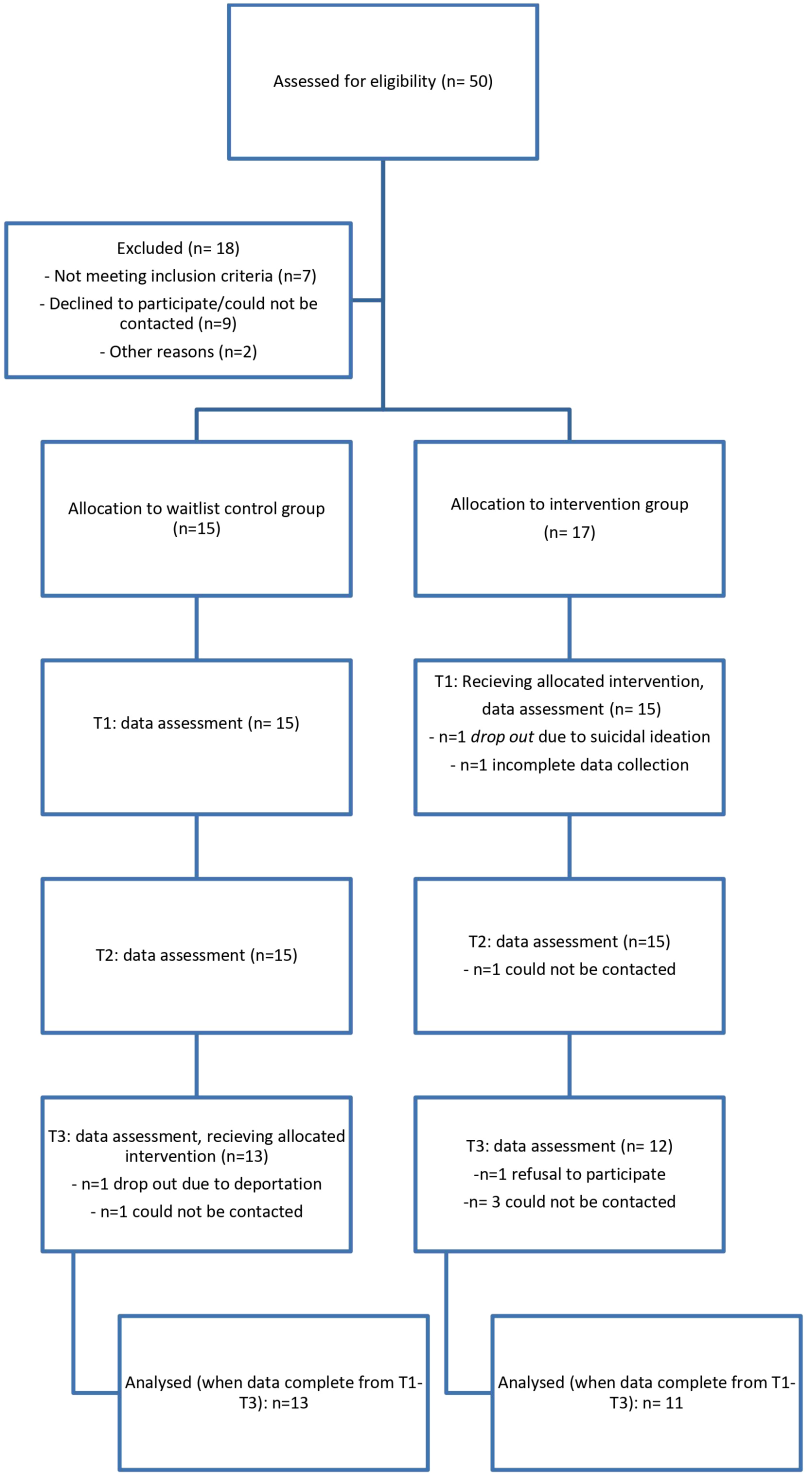
Flow chart for study participation.

From September 2020 until January 2021, *n*=190 refugees were contacted of which *n*=106 participated in an initial screening with screening questionnaires of symptoms of PTSD (PC-PTSD-5 (28)), depression, and anxiety disorder (PHQ-4 (45)). The results of this screening can be found elsewhere (46). 47,2% (*n*=50) fulfilled the cut off score of the initial screening questionnaire for post-traumatic stress disorder. Of the *n*=50 eligible participants, *n*=7 could not be included due to the language barriers. *N*=3 said that they were not interested in the study, one person was deported, and another person was transferred to another accommodation. Despite agreeing to participate, *n*=6 persons could no longer be reached for the initial appointment. *N*=32 agreed to take part in this study. In total, *n*=24 participants could be reached for all times of the data collection, which corresponds to a dropout rate of 25,0%. Detectable effect sizes of f=.27 were found using the software G-Power (43) for mixed ANOVA with α=.05, 1−β =.80, *n*=24, number of groups =2, number of measurements =3, corr. among rep measures =0.5, and non-sphericity correction ϵ=1 which indicates that medium effects can be found with our sample. Details of the study participation can be found in **Figure 1** (flow chart).

In this sample, participants were between 21 and 53 years old (M=33,8), 53,12% were male. They came from 12 different countries. At T1 68,75% of participants had already conducted their interviews on the asylum procedure and 46,87% were still waiting for their decision on their residency status (**Table 1**).

**Table 1:** Sociodemographic of participants (N=32).

Gender		
Male	N=17	53,12%
Female	N=15	46,87%
Age		
Mean	33,82	
SD	8,7	
Range	21-53	
Country of Origin		
Turkey	N=6	18,75%
Cameroon	N=3	9,37%
China	N=1	3,12%
Iran	N=7	21,87%
Gambia	N=3	9,37%
Togo	N=1	3,12%
Guinea	N=2	6,25%
Afghanistan	N=4	12,50%
Armenia	N=1	3,12%
Morocco	N=1	3,12%
Nigeria	N=2	6,25%
Algeria	N=1	3,12%
Asylum interview conducted		
Yes	N=22	68,75%
No	N=8	25,0%
Not specified	N=2	6,25%
Information on residency status recieved		
Yes	N=12	37,5%
No	N=15	46,87%
Not specified	N=5	15,62%

*N*=17 participants were randomly allocated to the intervention group, *n*=15 to the waitlist control group. Baseline clinical characteristics at T1 for both groups can be found in **Table 2**. There were no significant differences in both groups in relation to symptom severity in PTSD (t(28) = -.316 p= .754), depression (t(24,043) = -.107, p = .916), anxiety disorder (t(29) = -.215, p = .832) or perceived stress (t(29) = -.186, p = .854) at the start of the study.

**Table 2:** Baseline clinical characteristics at T1.

	Intervention group (n=17)	Control group (n=15)	t	df	p
PC-PTSD-5	3,95 (SD 0,89)	3,83 (SD 1,02)	-.316	28	.754
PHQ-2	3,87 (SD 2,41)	3,80 (SD 1,37)	-.107	24,043	.916
GAD-2	3,81 (SD 2,10)	3,66 (SD 1,63)	-.215	29	.832
Stress Thermometer	6,94 (SD 2,69)	7,73 (SD 3,39)	-.186	29	.854

For the following calculations, the data of the participants who participated in all data collection points (*n*=24) were included. There were only a few non-normally distributed variables (*p* <.05). Since the mixed ANOVA is relatively robust against the violation of the normal distribution, this procedure was continued (47). When calculating the depression symptoms and the item “arousal” of the SAM questionnaire, the variances were unevenly distributed. However, according to Bortz (48), the analysis of variance is relatively robust for samples of equal size and *n* > 10.The statistical analysis was carried out to evaluate the changes in PTSD, depression and anxiety symptoms as well as the perception of stress and emotional state over the course of the three measurement times. There was neither a statistically significant interaction effect for the change in PTSD symptoms with regard to time and group allocation (time*group (F(1,775,39,061) = .221, p = .777, η² = .010)), nor for depression symptoms (time*group F(2,44) = .047, p = .955, η² = .002) or symptoms of anxiety (time*group F(2,42) = .478, p = .624, η² = .022). With the help of the RHS thermometer as well as the SAM, the perceived stress and emotional state were recorded at all three measurement points. There was no significant interaction effect for the stress level (F(2,44)=.166, p=.848, η²=.007). There was also no significant interaction effect for emotional well-being (valence: Time*group: F(2.44)=.166, p=.848, η²=.007, dominance: Time*group: F(2,44)=.209, p=.812, η² =.009). The condition of equality of the covariance matrices is not given according to the box test (p=.029), thus the effect of the variable “arousal” cannot be interpreted (49). The results can also be found in **Table 3**. This study posed no harm to participants. However, one participant dropping dropped out at T1 due to suicidal ideation which became evident through the screening.

**Table 3:** Results of the mixed analysis of variance.

Measure	time				time x group			
	df	F	p	η²	df	F	p	η²
PC-PTSD-5	1,775, 39,061	.528	.573	.023	1,775, 39,061	.221	.777	.010
PHQ-2	2, 44	3,188	.051	.127	2, 44	.047	.955	.002
GAD-2	2, 42	1,532	.228	.068	2, 42	.478	.624	.022
Stress Thermometer	2, 44	.573	.568	.025	2, 44	.166	.848	.007
SAM - valence	1.755, 35.103	4.713	.019	.191	2, 44	.166	.848	.007
SAM - dominance	2, 44	.209	.812	.009	2, 44	.209	.812	.009
SAM – arousal**	2, 40	.324	.725	.016	2, 40	.952	.395	.045

*p<0.05.

** effects cannot be interpreted due to the inequality of the covariance matrices according to the box test (p=.029).

Regarding the questionnaire about the use of the exercises, participants indicated that within the period between T1 and T2, which was around four weeks, they used the exercises on average 8,25 times (Range= 0-30, SD = 11,04). Five participants indicated to not have used the exercises at all, two participants used it between one and 10 times and five participants stated, that they used the exercises more than 10 times within the study period. If the participants had used the exercises at least once, they were asked to indicate their personal assessment of the exercises on a visual analogue scale (1-10). Among those, who used the exercises at least once (*n*=7) positive effects while doing the exercises were reported (M = 7,88, SD = 1,76, Range = 5,3-9,8). Further, participants stated that they want to use the exercises in the future (M = 9,58, SD= 0,48, Range = 8,9-10.0).

## 4 Discussion

The aim of the current intervention study was to evaluate the feasibility of providing audio-based mindfulness and guided imagery techniques for traumatized refugees living in temporary accommodations. A further goal was to assess the effect of the intervention on the symptoms of post-traumatic stress disorder as well as depression and anxiety disorder, emotional well-being and perceived stress when used regularly on their own. The effects on the measured symptom burden compared to a comparison group did not show significant effects, if a medium effect size is assumed. It is of significant relevance to put our study results in the context of the current literature. There is a gap between the knowledge about an effective treatment of trauma-related disorders (50), and the question of how those refugees that are affected can be reached (51, 52).Various challenges and lessons learnt during the implementation of the study are discussed in the following discussion. These insights might ease and support further research in the field of refugee mental health.

First, one relevant challenge was to establish a careful approach in addressing the refugees in a suitable way. In some accommodations, social workers were always present to help establish contact, but in other accommodations, this was not the case. Here, the study team visited the accommodations on different days of the week at different times, and used written information about the study in order to contact as many people as possible. In the course of the screening, a high proportion of Kurdish-speaking refugees were living in the accommodation became evident. This was a language which could not be covered by the language offer. It also became clear that children were included in the information about the average number of residents provided by the local authorities, which was not considered before.

Regarding the PTSD prevalence in the research sample, the screening revealed, that out of *n*=106 adult refugees, 47.2% reached the cut-off value for a post-traumatic stress disorder. These results confirm high prevalence of PTSD in refugee population. Although the sensitivity (0.95) and specificity (0.85) of this screening questionnaire is high (28), it should be noted that other studies have consistently found a higher prevalence with self-report questionnaires than with external assessment of PTSD symptomatology (1). In the present study, the refugees were approached by the study team in their accommodation. From the perspective of the people concerned, this entails the difficulty of talking to strangers about the topic of mental health. A variety of factors, like fear of stigmatization, addressing taboo topics (53), uncertainty about what is considered “normal” stress or an illness-related symptom (54, 55) may cause the person being contacted in a one-time conversation in the shelter to refrain from the opportunity to take part in the study even though they are mentally burdened. In this case, greater involvement of a person of trust, such as local social workers on sight, might have been a way to better reach those affected.

Autonomous practice was another major difficulty, that became apparent in the process of the study: Only a few participants regularly used the exercises on their own. By offering self-application exercises in digital form and in the participants’ native languages, this approach aimed to overcome two major challenges in the psychosocial treatment of refugees: the language barrier and limited access to mental health care (7). The aim of the translation into six different languages was to make stabilizing exercises available to a large number of refugees. The advantages of providing audio files on smart phones include simplified availability and flexibility of use in their everyday life. Although the present study did not systematically record why the exercises were not used, various reasons became clear throughout the study. A period of 4 weeks seemed to be too short to establish regular use. After the time of intervention was over, few participants approached the study team to ask about the exercises again. However, it also seemed that some participants benefited from being repeatedly asked and encouraged to use the exercises.

In the study by Röhr et al. (2013), the authors evaluated a self-help App which was designed for the use by Syrian refugees suffering from PTSD. The authors similarly described, that 30% of their participants never used the App. Further, they pointed out, that potentially motivating calls by the study team could have enhanced the use (27). Regular text message reminders could potentially enhance adherence to the exercises. The use of the app from created by Mazzulla et al. (26), was introduced in the form of several group meetings, which possibly could have positively affected to the weekly use of the app by all participants. In the qualitative interviews conducted with the participants of the group sessions by Zehetmair et al. (2019). The lack of support from both group members and the therapist was reported. This lack was identified as a hindrance to integrating the exercises into everyday life. The participants in this study had not yet been able to draw upon positive experiences with the exercises in a group setting to develop their own motivation for using them. It is possible that a more established group environment, as seen in the pilot study, might have provided greater opportunities for participants to engage with the exercises, fostering motivation and contributing to symptom reduction. Moreover, common therapeutic factors, such as regular empathetic contact, group support, and the shared experience of not facing problems alone (56, 57), could have potentially encouraged sustained exercise use and alleviated symptoms, but they would have not been suited to the study. Within this context, the establishment of a group setting in turn would have led to the offer being limited to one or a few languages and increased personnel costs. It should also be noted that in the conversations with the participants of the present study, many of them stated that they were very concerned with post-migration stress factors, such as the asylum process, obtaining a work permit or finding an apartment. In the pilot study in the group setting, the stressors of daily life were also cited as an obstacle, which is why the participants did not use the exercises regularly (23).

The challenging living conditions faced by refugees, combined with their frequent exposure to traumatic experiences (58) contribute to a significant overall burden. The participants consisted of refugees who were still in a legally uncertain situation and lived in shared accommodations. In addition, family separation (59) and unemployment (60), which was often the case for the participants, are factors that have been repeatedly shown to be associated with more severe symptom burden (61, 62). This high level of burden may explain the lack of statistically significant results in the study, suggesting that, given the life circumstances of the participants, the chosen intervention may not have been sufficient to produce a substantial impact. Trauma-confrontative therapy approaches, which have been proven to reduce the symptom burden of PTSD (50), were also not carried out in this study. Further, interventions for this population may also have to counteract the effects of post-migration stressors in order to achieve a significant reduction in symptom burden. This aspect has been included for example in a major WHO project, evaluating a transdiagnostic approach “Problem Management Plus”, which targets general stress management and problem-solving strategies and behavioural activation (63–65).

In conclusion, with respect to the specific objectives of the study, providing imagination exercises within a limited time frame and without regular (therapeutic) contact led to independent use by only a minority of the participants. While small effects or symptom improvement in individuals cannot be ruled out, such outcomes may not be detectable given the sample size. However, the significance of the study also stems from the challenges encountered in establishing initial contact with individuals in temporary accommodations, addressing their psychological distress, and providing support that is both effective and feasible within the constraints of the described circumstances. In these living conditions, the effectiveness of social support in parallel with psychological therapy approaches should possibly be examined. The existing structures, such as the presence of social workers and, in non-corona times, volunteers, as well as the interpersonal connections between people, should be combined. These could be some strategies for addressing the challenges stated here (52).

## 5 Limitations

Various methodological constraints need to be mentioned. Individuals who consistently engaged in the exercises may have experienced symptom improvement, though the small sample size limits the ability to detect smaller effects. As a result, only medium effects of the intervention could have been observable. Additionally, the sample size and limited participation by refugees restricted the statistical evaluation and lowered the generalizability of the findings to the broader population of asylum seekers. Further, the PC-PTSD-5 was used as a tool to identify and include possible participants. This cannot replace a structured clinical interview (66) for a final diagnosis. Moreover, the data collection was kept low-threshold with few item numbers. This was done due to the diverse challenges in terms of language, approaching people to participate in the study and living conditions, to reach as many affected people as possible. However, this limits the preciseness of the results. In addition, it should be noted that the study was carried out during the Covid19 pandemic, which, on the one hand, led to increased difficulties in establishing contact and data collection (e.g. due to quarantine). On the other hand, it cannot be ruled out that this may have had an additional negative impact on the psychological burden of the refugees (67).

## Data Availability

The raw data supporting the conclusions of this article will be made available by the authors, without undue reservation.
