# Supplementary material for: Implementing a stabilizing intervention for traumatized refugees in temporary accommodations in South-West Germany - a randomized controlled pilot trial
Source: Front Psychiatry. 2024 Oct 31;15:1453957. doi: 10.3389/fpsyt.2024.1453957 (PMC11560747; doi:10.3389/fpsyt.2024.1453957)
Supplement: Supplementary file 1 [file Table1.docx]

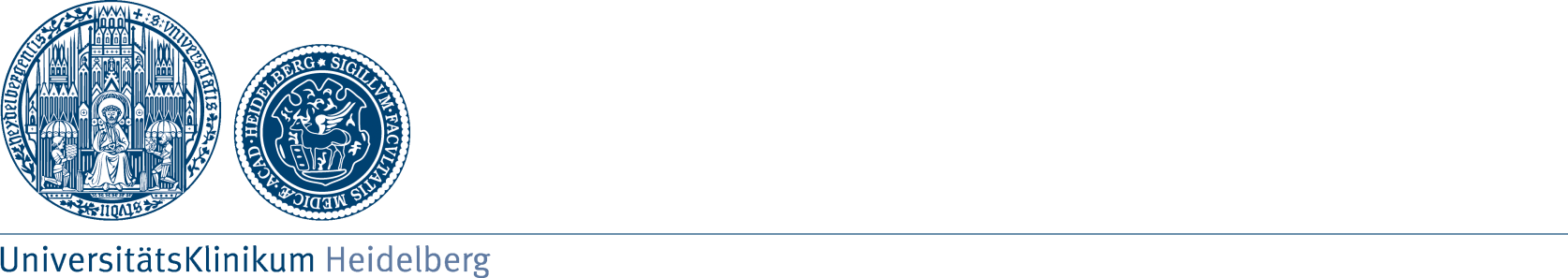


**“Support Offers for Refugees with Post-traumatic Disorders”**

**Informed consent - study participant**

I (me), ___________________________________ (Last name, First name) herby give my consent – which I may withdraw at any time – to participate in the following study called „**Support offers for refugees with post-traumatic disorders“**. I have had the nature, significance, and risks of the scientific investigations carried out as part of the above study explained to me both orally and in writing via the information sheet. I have had the opportunity to ask questions. All my questions were answered to my satisfaction. I voluntarily agree to participate in the study. I had sufficient time for my decision. I have received a copy of the information sheet and the informed consent.

Code

| First letter of your first name | First letter of your last name | Numeric code of the investigator |
| --- | --- | --- |

**Data Privacy**

I am aware that personal data will be processed in this study. The data will be processed in accordance with legal requirements and, pursuant to Art. 6 para. 1 lit. of the Basic Data Protection Regulation, requires the following declaration of consent:

I have been informed and voluntarily agree that my data collected in the study, in particular information about my health^[[1]](#footnote-1)^, can be recorded and evaluated in pseudonymised form for the purposes described in the information document. Third parties will not be given access to personal documents. My name will also not be mentioned when the results of the study are published. The data will be kept for up to five years after the end of the study. I am aware that this consent can be revoked at any time in writing or verbally without giving reasons, without any disadvantages for me. This does not affect the legality of the data processing that took place until the revocation. In this case, I can decide whether the data collected by me should be deleted or whether it may continue to be used for the purposes of the study.

To ensure organization of the study to a later time of measurement my contact details are required.

My e-mail Address: _________________________________

My cellphone number: _________________________________

, _______________ _______________________________________

(Signature of the participant)

_______________________________________

(NAME OF THE PARTICIPANT IN BLOCK LETTERS)

Enlightening person

The patient was informed by me in a conversation about the aim and the course of the study as well as the risks. I gave the patient a copy of the information sheet and the informed consent.

, _______________ _______________________________________

(Signature of the investigator)

_______________________________________

(NAME OF INVESTIGATOR IN BLOCK LETTERS)

1. According to Art. 9 para 1 DSGVO health data are personal data of a special category which the study participant must explicitly consent to process. The same applies to data revealing racial or ethnic origin, political opinions, religious or philosophical beliefs or trade union membership, as well as to the processing of genetic data, biometric data uniquely identifying a natural person, data concerning sexual life or sexual orientation. [↑](#footnote-ref-1)
