# Supplementary material for: Implementing a stabilizing intervention for traumatized refugees in temporary accommodations in South-West Germany - a randomized controlled pilot trial
Source: Front Psychiatry. 2024 Oct 31;15:1453957. doi: 10.3389/fpsyt.2024.1453957 (PMC11560747; doi:10.3389/fpsyt.2024.1453957)
Supplement: Supplementary file 2 [file Table2.docx]

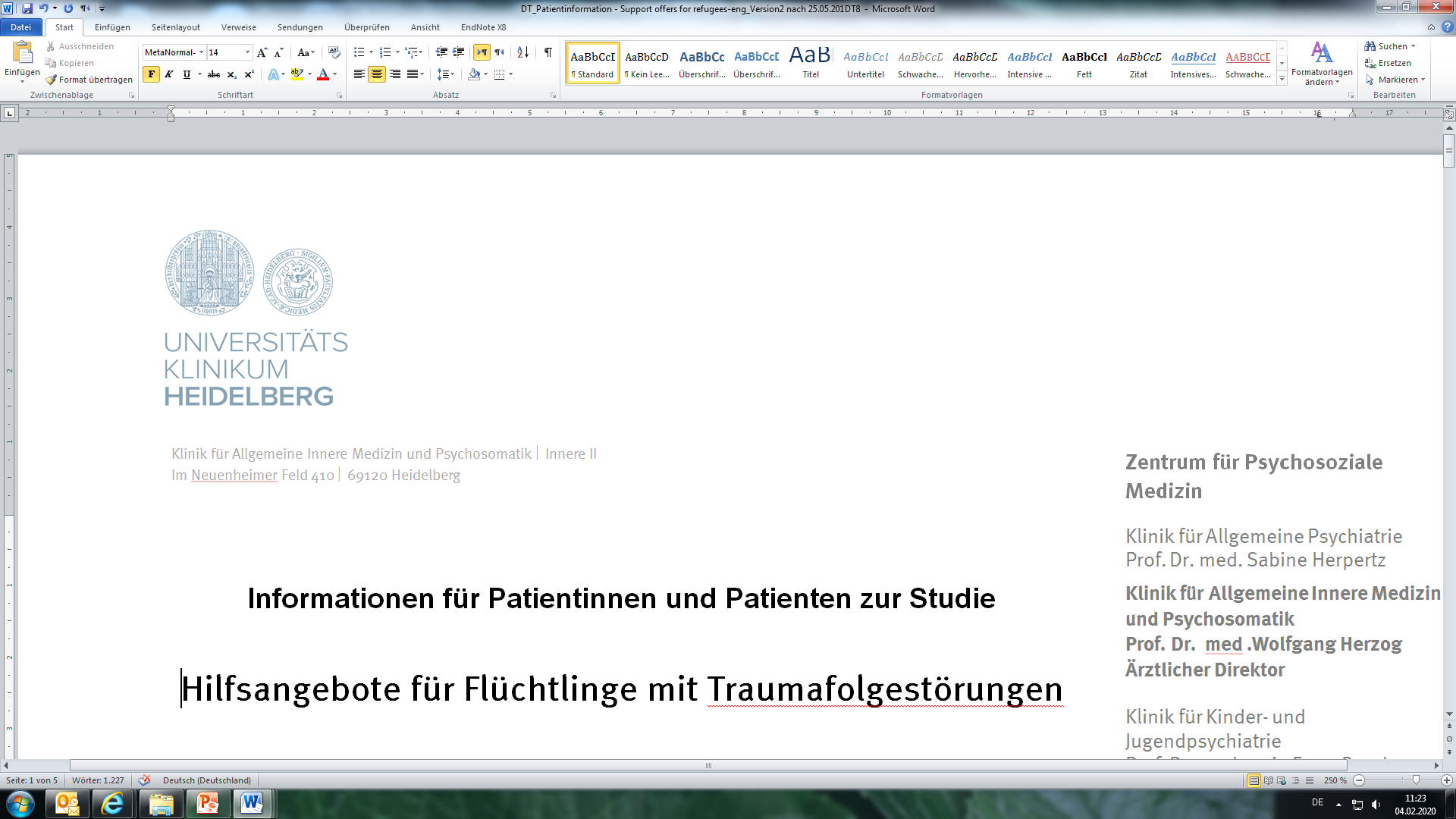


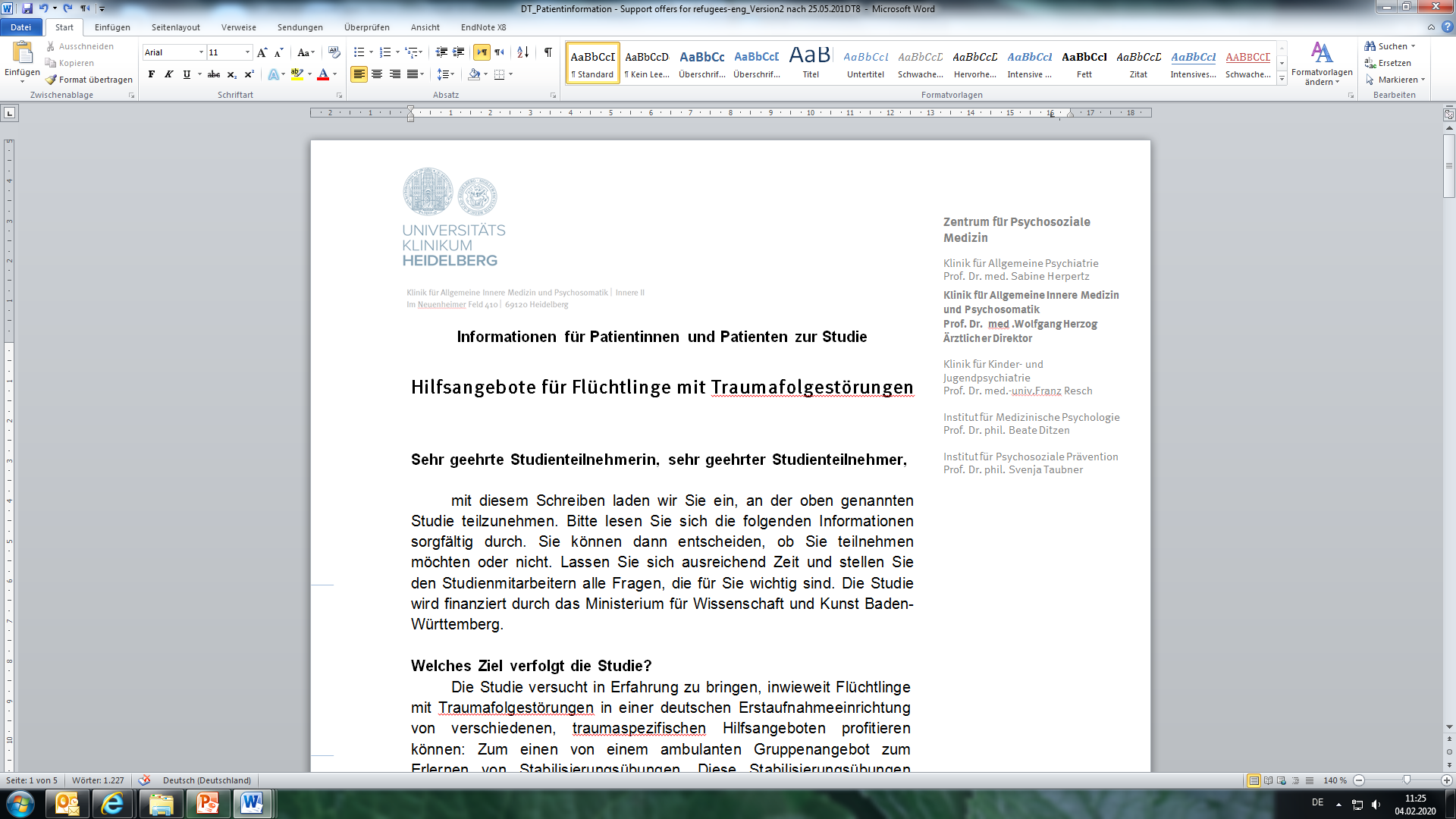


**Patient Information Fact Sheet**

**“Support Offers for Refugees with Post-traumatic Disorders”**

**Dear Sir or Madame,**

in the following, we would like to inform you about a research study which might be of interest to you. We would be pleased to convince you to participate in the study. The study is called “**Support offers for refugees with post-traumatic disorders**”. Before you decide whether you would like to participate, you need to understand why the research is being done and what it would involve for you. Please take time to read the following information carefully. Please ask us if there is anything that is not clear or if you would like more information. We will be happy to discuss your questions with you in detail. The study will be supervised by (apl.) Prof. Dr. Christoph Nikendei, MME (Internal Medicine II) and Catharina Zehetmair (also Internal Medicine II).

**What is the purpose of the study?**

In this study, we would like to investigate to which extent refugees with post-traumatic disorders can benefit from different trauma-specific support offers in a German (primary) care facility. Firstly, the study will investigate the benefits of an outpatient group teaching stabilization techniques. These stabilization techniques aim to help participants control their physical and psychological arousal more effectively and to self-soothe, while also enabling them to stop and / or gain better control over unpleasant flashbacks. **Secondly, the study will investigate whether participants perceive the opportunity to regularly practice stabilization techniques at home by themselves with audio instructions as beneficial.** And thirdly, we would like to examine whether participants are able to benefit from taking part in an "Eye Movement Desensitization and Reprocessing" (EMDR) outpatient group. EMDR is a specific psychotherapeutic method which is helpful for the processing and integration of traumatic experiences as well as for the treatment of trauma associated fears.

As part of this process, you may be asked some of the following questions: How was that for you? Are you able to use the learned stabilization techniques or elements of the EMDR course? Do the techniques help you improve your physical rest and peace of mind? Furthermore, we will also ask you to evaluate the support offers you took part in: How satisfied were you with the outpatient group and the opportunity to practice the techniques at home by yourself? Were the offers helpful?

**What will happen if I take part?**

If you would agree to take part in the study, you will be assigned to one of three settings in a first step: In setting one (stabilization group), participants will start their first session in a timely manner, during which they will learn various stabilization techniques and practice them. A total of twelve sessions of 60 minutes will be offered on a weekly basis. **In setting two (self-governed stabilization), participants will be given various stabilization exercises as audio files and will be encouraged to practice them at home by themselves in the coming weeks.** Participants in setting three (EMDR group) will also start with their first session promptly, during which they will learn and practices EMDR techniques. Again, a total of twelve sessions of 60 minutes will be offered on a weekly basis. Following each group meeting, we will ask all participants in the stabilization and EMDR group to complete a questionnaire, which will take approximately 20 minutes. Participants in the self-governed stabilization setting will receive the questionnaires in advance and will be asked to complete one of the sheets each week. The questionnaires will ask questions about the severity of different symptoms, on the one hand, and ask you to evaluate your satisfaction with the support offer you are taking part in, on the other hand.

After the last meeting, we would like to invite you to take part in a 30 minute interview. If you agree to participate, will ask you for your personal feedback on the support offer you took part in. The interview will be recorded on tape. The audio recording will be analyzed by scientific staff from the Heidelberg University Hospital.

**What will happen to my interview tape and data?**

All the information you give us will be treated with strict confidentiality. No personal data (such as name, date of birth, address, background of migration, or other information that allows conclusions to be made about your person) will be passed on to third parties.

We would be very pleased if you would agree to participate in the study and audio recording of the interview.

**What are the possible benefits and risks of taking part?**

Through your participation, you will make an important contribution toward finding out whether or not refugees with post-traumatic disorders can benefit from learning stabilization techniques in the short term. In addition, your participation as well as your feedback will help us evaluate the provided support offers. The results of this study form the basis for the development of further support offers to improve the situation of refugees with traumatic disorders. If you are interested, you can also receive individual feedback after we have evaluated the data. If you would like to receive personal feedback, please contact M.sc.-Psych. Catharina Zehetmair (telephone: 06221/56-8373; Mail: [Catharina.Zehetmair@med.uni-heidelberg.de](mailto:Catharina.Zehetmair@med.uni-heidelberg.de)).

There are no risks associated with taking part in this study’s outpatient group support offer teaching stabilization techniques, nor in completing the questionnaires and talking about the support offers afterwards or in the use of the stabilization exercise audio files.

**What if I don’t want to take part in this study, or if I want to withdraw later?**

Participation in this study is voluntary. It is completely up to you whether or not you participate. You can withdraw your consent at any time in writing or orally without giving reasons and without any disadvantage for you. If you wish to withdraw from the study, we will destroy all your collected data, if you wish. Should you wish to withdraw from the study at a later date, please contact M.Sc.-Psych. Catharina Zehetmair (telephone: 06221/56-8373; Mail: [Catharina.Zehetmair@med.uni-heidelberg.de](mailto:Catharina.Zehetmair@med.uni-heidelberg.de)).

**Will my taking part in the study be kept confidential?**

Medical confidentiality and data protection regulations are observed. All the information you give us will be treated with strict confidentiality. During the study, your personal information will be collected and stored in your personal file at the University Hospital of Heidelberg, some of which will be stored electronically. The data important for the study are additionally stored and evaluated in pseudonymised^[[1]](#footnote-1)^ form. The study management will take all reasonable steps to ensure that your data is protected in accordance with the data protection standards of the European Union. The data is secured against unauthorized access. A decryption will only take place if you withdraw from the study for the purpose of data destruction. As soon as possible after the research or statistical purpose, the personal data will be anonymised^[[2]](#footnote-2)^. The data collected during the study will be kept until the data evaluation is completed. The data will be used exclusively for the purposes of this study.

You have the right to request information from the person responsible (see below) for the personal data stored about you. You can also demand the correction of inaccurate data and the deletion of the data or restriction of its processing.

**Who do I contact for further information?**

| **Project coordinator** | **Lead investigator** |
| --- | --- |
| M.sc.-Psych. Catharina Zehetmair  [Catharina.Zehetmair@med.uni-heidelberg.de](mailto:Catharina.Zehetmair@med.uni-heidelberg.de)  Clinic for General Internal Medicine and Psychosomatics  University Hospital Heidelberg  Thibautstrasse 4  69115 Heidelberg | Prof. Dr. med. Christoph Nikendei, MME  [Christoph.Nikendei@med.uni-heidelberg.de](mailto:Christoph.Nikendei@med.uni-heidelberg.de)  Clinic for General Internal Medicine and Psychosomatics  University Hospital Heidelberg  Thibautstrasse 4  69115 Heidelberg |

If you have any concerns regarding data processing and compliance with data protection requirements, you can contact the following data protection officer at the institution:

Data protection officer of the University Hospital of Heidelberg

Im Neuenheimer Feld 672

69120 Heidelberg

06221-567036

[datenschutz@med.uni-heidelberg.de](mailto:datenschutz@med.uni-heidelberg.de)

In case of unlawful data processing, you have the right to complain to the following supervisory authority:

The State Commissioner for Data Protection and Freedom of Information Baden- Württemberg

PO Box 10 29 32, 70025 Stuttgart

Königstraße 10a, 70173 Stuttgart

Tel.: 0711/61 55 41 – 0

Fax: 0711/61 55 41 – 15

E-Mail: poststelle@lfdi.bwl.de

Internet: http://www.baden-wuerttemberg.datenschutz.de

**Will taking part in this study cost me anything, and will I be paid?**

Participation in this study will not cost you anything.

**Weitere Informationen**

Für weitere Informationen sowie für Auskünfte über allgemeine Ergebnisse und den Ausgang der Studie steht Ihnen als Leiterin der Studie Frau Catharina Zehetmair (Telefon: 06221 56-8373, Email: Catharina.Zehetmair@med.uni-heidelberg.de) zur Verfügung.

**Thank you for considering taking part in this research!**

**If you wish to take part in it, please sign the attached consent form.**

**This information sheet is for you to keep.**

1. „Pseudonymization“ means the encryption of personal data in such a way that the personal data can no longer be assigned to a specific person without the need for additional information ('key'). This additional information is stored separately and is subject to technical and organizational measures that ensure that the personal data is not assigned to an identified or identifiable natural person. [↑](#footnote-ref-1)
2. "Anonymisation" means the alteration of personal data in such a way that the data subject can no longer be identified or can only be identified at a disproportionate cost or time. [↑](#footnote-ref-2)
